# Acute Diarrhoea in Children: Determination of Duration Using a Combined Bismuth Hydroxide Gel and Oral Rehydration Solution Therapy vs. Oral Rehydration Solution

**DOI:** 10.3390/children3040045

**Published:** 2016-12-21

**Authors:** Adriana Oviedo, Mirna Díaz, María Laura Valenzuela, Victoria Vidal, Liliana Racca, Hebe Bottai, Graciela Priore, Graciela Peluffo, Susana Di Bartolomeo, Graciela Cabral, María del Carmen Toca

**Affiliations:** 1 Sección de Gastroenterología, Servicio de Pediatría, Hospital Nacional Alejandro Posadas, 1684 El Palomar, Buenos Aires, Argentina; adriana-oviedo215@hotmail.com (A.O.); mldiazs@gmail.com (M.D.); lauravalenzuelavera@gmail.com (M.L.V.); vickyeugevidal@hotmail.com (V.V.); 2 Área de Estadística y Procesamiento de Datos, Facultad de Ciencias Bioquímicas y Farmacéuticas, Universidad Nacional de Rosario, 2000 Rosario, Santa Fe, Argentina; lracca@fbioyf.unr.edu.ar (L.R.); hbottai@fbioyf.unr.edu.ar (H.B.); 3 Sección de Microbiología, Laboratorio Central, Hospital Nacional Alejandro Posadas, 1684 El Palomar, Buenos Aires, Argentina; gracielaleonorpriore@gmail.com (G.P.); peluffo.graciela@gmail.com (G.P.); sudiba@gmail.com (S.D.B.); 4 Sección Virología, Laboratorio Central, Hospital Nacional Alejandro Posadas, 1684 El Palomar, Buenos Aires, Argentina; gracielacabral@yahoo.com

**Keywords:** acute diarrhoea, bismuth hydroxide gel, hydration salts

## Abstract

Oral rehydration salt (ORS) treatment in young children with acute diarrhoea (AD) has contributed to decrease mortality associated with dehydration although effective strategies to reduce morbidity associated with this disease are required. The aim of this study was to evaluate the diarrhoea duration when using combined colloidal bismuth hydroxide gel (CBHG) and oral rehydration salt treatment compared with ORS therapy in children with AD. We designed a double-blind, randomised prospective study with treatment and control groups. Patients aged one to 12 years, with no prior pathology and with AD of less than 48 h were included. The Chi-squared and Mann-Whitney tests were used, as well as the Cox proportional hazards model and the Kaplan-Meier estimator. Patients were randomised into an ORS and CBHG treatment group and a control group for ORS plus placebo. (Average age: 3.2 years). The result of the post-treatment evaluation with respect to the average duration of AD was 25.5 h for the treated group vs. 41.5 h for the control group (*p* = 0.015). The average number of stools was 4.8 in the treated group and 8.2 in the control group (*p* = 0.032). We conclude that the use of CBHG plus ORS significantly reduced the duration of AD, the number of stools and the percentage of children with persistent AD after 24 h of treatment compared to the control group. AD remitted almost twice as fast in patients treated with CBHG and ORS compared to those who received ORS plus placebo.

## 1. Introduction

The World Health Organization (WHO) defines acute diarrhoea (AD) as three or more watery, liquid or semi-liquid stools over a 24-h period. The change from the normal pattern of bowel movements is the most important clinical information, based on changes in the frequency and consistency of the stools. It usually lasts less than seven days but may be as long as 14 [1,2]. Nausea, vomiting, fever, abdominal pain and general malaise may accompany AD. The possible electrolytic and nutritional consequences are responsible for its morbidity and mortality [2]. In healthy children, it is generally a self-limiting disease, whereas it can be prolonged in undernourished children. In addition to the frequency and volume of the stools, vomiting must be considered an indirect indicator of increased risk and severity [3].

The Boletín Integrado de Vigilancia 2012 of the Ministry of National Health on the epidemic of diarrhoea in Argentina states that the most frequently identified bacteriological agents are *Shigella* spp., specifically *Shigella flexneri*; *Escherichia coli*, predominantly enteropathogenic *E. coli*, and *Salmonella* spp. For viral diarrhoea, the greatest quantity of positive cases are due to rotavirus, which accounts for more than 90% of positive viral cases found in outpatients and inpatients, with an overall positivity of approximately 30%, and which varies considerably throughout the year [3]. The seriousness of clinical symptoms is related to dehydration, for which reason in severe cases it becomes a priority to determine changes in intravascular volume and electrolyte disorders before identifying the causative organism.

The WHO highlights the safety and effectiveness of therapy involving oral rehydration salts, and emphasizes the importance of adequate fluid, electrolyte and nutritional management as keys to its treatment [4,5].

Nevertheless, today diarrhoea continues to be an important cause of morbidity and mortality in small children. It is therefore necessary to find effective strategies for reducing the morbidity associated with this disease.

Colloidal bismuth hydroxide gel (CBHG) is an antisecretory drug that has been used for many years for treating and preventing AD and traveller’s diarrhoea, as well as against *Helicobacter pylori*. CBHG interferes in the pathogenesis of AD via various mechanisms: it promotes and restores the integrity of the mucous membrane, it is not absorbed, it acts in the intestinal lumen without inhibiting peristalsis, thanks to its antisecretory effect and the fact it inhibits virulence factors it can be an alternative as an adjunctive therapy [3]. Recent studies have shown that it directly influences gastrointestinal pathogens: in vitro it inhibits the replication of human rotavirus and bacterial virulence factors, including the activity of Shiga toxins [6,7,8,9,10,11,12,13,14].

CBHG is not a salt, so it contains no associated radicals such as salicylate, which is responsible for the adverse effects and contraindications of bismuth subsalicylate [11].

These mechanisms suggest that CBHG could be useful both in preventing and reducing the seriousness and duration of AD.

Using bismuth as CBHG together with an oral rehydration solution (ORS) appears to be an alternative for treating AD under the hypothesis of complementing hydration with a therapy that acts directly on reducing the duration of the diarrhoea and quantity of stools.

## 2. Materials and Methods

A double-blind, randomised prospective study was undertaken, with a treatment group and a control group.

The present study was evaluated, approved and authorized for implementation, and registered by the Bioethics Committee “Dr. Vicente Federico Del Giúdice”, the clinical research protocol evaluation commission of the Hospital Nacional Profesor Alejandro Posadas (Supplementary data 1). 

Between 2013 and 2015, children aged one to 12 years with AD of less than 48 h of evolution were included in the study.

The sample size calculation was established as 25 patients who concluded the treatment in each group, considering a power of 0.80 with a significance level of 0.05.

To compare the two groups prior to treatment, the Chi-squared test was applied for the sex, vomiting and fever variables, and the Mann-Whitney test was used for age and number of stools passed. The post-treatment comparison of diarrhoea duration and number of stools was also undertaken using the Mann-Whitney test, whereas Fisher’s exact test was selected for the vomiting and fever variables.

The assessment of normality adjustment for the variables was made for the quantitative variables: age, number of previous stools, duration of the diarrhoea, and number of stools during that period. Since the variables did not show a normal distribution, a non-parametric test was applied (Mann-Whitney test).

Normality was proven graphically as well as by using the Shapiro-Wilk test. All the *p*-values were less than 0.005, except for the number of previous stools, which was 0.37 in the treated group only.

The patients were only included once the father and/or mother had been informed about the study in a way they could understand, they had comprehended this, and they had signed the approved version of the Informed Consent form (Supplementary data 2).

The inclusion criteria were: children of either sex, with non-dysenteric stools, no prior pathology, being eutrophic, normohydrated and the responsible relatives of whom understood and accepted the study. The exclusion criteria were established as: AD of longer than 48 h of evolution, the existence of a prior pathology, a diagnosis of dysentery, patients who needed hospitalization, the non-acceptance of the treatment by the family and the non-comprehension of the informed consent.

No patient received any per diem allowance for participating in the study.

When the patients were admitted, the variables of sex, age, presence of vomiting, febrile state, and type and number of stools were recorded for the 24 h prior to the consultation. The nutritional state of the child and their hydration was evaluated, and a stool culture, virological study and parasitological analysis of fresh faecal matter were requested.

The treatments were randomised using the Random Number Table of Dawson-Saunders and Trapp (1997) [15].

In the first consultation, the treatment each patient received was completely numbered and randomised. The treatment given corresponded to the patient’s recruitment number. The control group received ORS plus a placebo; the treatment group received ORS plus GHBG (Crema de Bismuto Chobet^®^, Laboratorio Soubeiran Chobet, Buenos Aires, Argentina , certificate no. 30,223, according to the instructions and doses approved by the National Administration of Medicines, Foods and Medical Technology (ANMAT, Administración Nacional de Medicamentos, Alimentos y Tecnología Médica)). The preparation of the ORS and how to administer it was explained to the father and/or mother of each patient in line with the Guidelines for Prevention and Treatment of Acute Diarrhoea of the National Directorate for of Motherhood and Infancy of the National Ministry of Health and all the children received a suitable calorie intake through a diet adjusted to their age and the standards for dealing with AD from the same ministry. The CBHG dose was indicated in line with the dosage approved by ANMAT.

Once the treatment had been initiated, all the patients were subject to daily control over a 7-day period (even though the diarrhoea had stopped), or until their stools returned to normal and the discharge of the patient in those cases that lasted longer than seven days. The frequency and number of stools, presence of vomiting, fever, and the appearance of adverse effects were all recorded. The duration of the diarrhoea was determined in hours from the beginning of the treatment until the stools returned to normal (less than three and with a normal consistency).

### Statistical Study

The Kaplan-Meier estimator was used to describe the percentage of children with persistent diarrhoea in both groups. Finally, a Cox proportional hazard model was applied to study the effect of sex, age and number of stools during the 24 h prior to the start of the therapy, and the treatment received, on the time the AD ended.

## 3. Results

**Figure 1:**
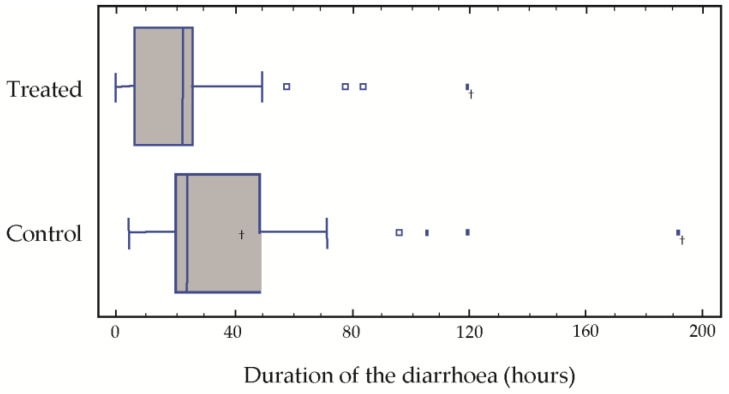
Duration of the diarrhoea for each sample group.

235 children were evaluated (Figure 1) who coincided with the spontaneous demand at the clinic of the Paediatric Service at the Hospital Nacional Alejandro Posadas, Buenos Aires, Argentina, and who had symptoms that could be managed with outpatient treatment. 119 cases were excluded as they did not fulfil the inclusion criteria: 104 referred more than 48 h after the onset of diarrhoea, 10 had dysentery symptoms, and five had other associated diseases.

The 113 patients who fulfilled the inclusion criteria were randomised into two groups: one group contained 58 children who received treatment with CBHG and ORS, and a control group with 55 children was given placebo and ORS.

63 patients completed the treatment and attended all the follow-up consultations: 29 from the treatment group and 34 from the control group. The other 50 did not attend subsequent consultations: 29 from the treatment group and 21 from the control group.

The treatment group included 20 boys and nine girls; and the control group had 16 boys and 18 girls. The median age in both groups was two years old (Table 1).

With regard to clinical symptoms, at the start of the disease, 23 patients from the treatment group and 25 from the control group presented vomiting, whereas four children from the treatment group and 10 from the control group displayed fever symptoms. The median number of stools was six (standard deviation of 2.7) and four (standard deviation of 2.0), respectively, for the two groups (Table 1).

It is necessary to point out that there were no statistically significant differences between the basal characteristics of the two groups with regard to sex, age, vomiting, fever and number of previous stools (Table 1).

The results of the post-treatment evaluation in our population with respect to the average duration of the diarrhoea were 25.5 h in the treatment group treated as opposed to 41.5 in the control group. Over the treatment period, the average number of stools was 4.8 and 8.2, respectively, in the two groups. Significant differences were found in the duration of the diarrhoea (*p* = 0.015) and the number of stools (*p* = 0.032) (Table 2). The medians of both the duration of the diarrhoea and the number of stools passed were significantly greater in the control group (Figure 1).

After 72 h of treatment, only three children of the treatment group presented diarrhoea, which was prolonged to 80, 90 and 120 h in these three cases. In the group control, four children presented symptoms of diarrhoea at 90, 110, 120 and 190 h, respectively (Figure 1).

**Figure 2:**
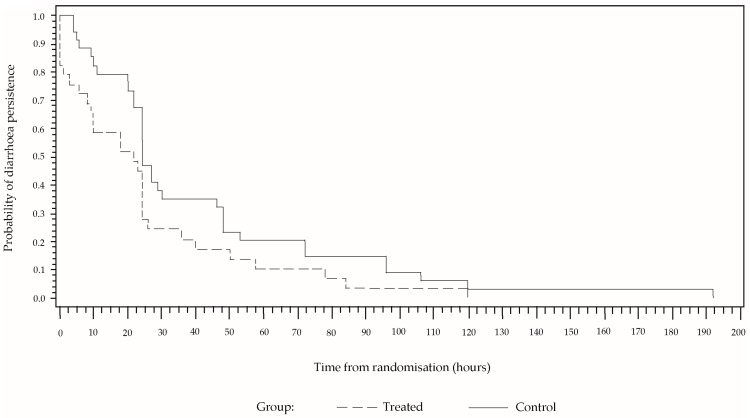
Kaplan-Meier estimator. Estimated cumulative percentage of children with persistent diarrhoea.

In a high percentage of cases, the diarrhoea reduced abruptly at the beginning and then decreased gradually until reaching a value of zero (Figure 2). Although the behaviour is similar in both sample groups, the percentage persistence of diarrhoea was greater in the group that received only ORS for the entire time, from the randomisation to the end of the treatment. In a Cox proportional hazard model including the covariables of sex, age and number of stools for the 24 h prior to the treatment, these covariables were not found to be significant, whereas the effect of the study group was significant (*p* = 0.0269). The estimated hazard ratio was 1.93 (CI 95% [1.4; 4.8]). After 24 h of treatment, diarrhoea was present in 27.6% of the cases in the group treated with CBHG and ORS, and in 47.1% in the control group. The number of children needed to treat is 5.13.

No patient presented either fever or adverse effects during the treatment. No significant differences were seen in the presence of vomiting and fever in the results obtained for the two groups. For the duration of the diarrhoea and number of stools over that period, the Mann-Whitney test was applied. Fisher’s exact test was employed to compare the presence of vomiting and fever (Table 2).

In all the cases, the AD was self-limiting and early feeding was tolerated. Also, no cases of prolonged diarrhoea or post-gastroenteritis syndrome were recorded.

## 4. Discussion

Although AD is a self-limiting disease in healthy children, its importance should not be underestimated, as it is known that children under five can have one or two annual events, generating multiple doctor visits and hospital stays. Over the last 20 years, incorporating ORS into the treatment, and early feeding guidelines, have significantly reduced the morbidity and mortality. The early cessation of diarrhoea is particularly important for the patient’s condition, since it avoids a greater loss of fluids and favours hydration. In the family environment, the more rapid reduction of diarrhoea involves the normalization of daily activities such as going to school and work.

Pharmacological treatment is not considered a cornerstone of AD therapy, however, as a measure for shortening the course of the disease, bismuth has been used for decades to treat diarrhoea. Recent studies have demonstrated its inhibitive power on the replication of rotavirus and bacterial virulence factors [6,7,8,9,10]. Its formulation as bismuth hydroxide avoids the adverse effects of salicylate [11].

The objective of this study was to determine the reduction in AD, for which reason the combined use of ORS and an antisecretory drug like CBHG was tested, under the hypothesis of complementing the absorption while diminishing the elimination of electrolytes and water. The CBHG acts in the gastrointestinal lumen, its absorption is negligible and it has no adverse reactions [11].

A population of healthy children in outpatient treatment was selected for the study. To accurately assess treatment response, it was decided to monitor the patients on a daily and longer-term basis. Although this made the study more rigorous, it led to the loss of a high percentage of patients from both groups (50% from the treatment group and 38% from the control group), which could correlate either with an improvement in the symptoms thanks to the effectiveness of the treatment or with the self-limiting character of AD.

Our results show a significant reduction in the duration of the diarrhoea and number of stools in patients who received the combined treatment of ORS and CBHG with respect to the group that received only ORS. After 24 h, only 27.6% of cases from the treated group still had diarrhoea as opposed to 47.1% in the group control.

In the patients treated with CBHG and ORS diarrhoea stopped at a rate almost double that of patients who took only ORS, meaning that in the treatment group the diarrhoea ceased almost twice as fast as in the control group. The number of children needed to treat is 5.13, i.e., approximately five children with AD should be treated with CBHG so that in one of them the diarrhoea does not persist after 24 h.

## 5. Conclusions

The combined use of CBHG and ORS significantly reduced the duration of the diarrhoea. Also, there was an important decrease in the number of stools and the persistence of the diarrhoea after 24 h of treatment with respect to the control group. Diarrhoea remission was almost twice as fast in the patients treated with CBHG and ORS in comparison with those that received only ORS. These results suggest the therapeutic use of a combined ORS/CBHG treatment contributes to improving the evolution of AD.

## Figures and Tables

**Table 1:** Basal demographic and clinical characteristics according to study group.

Characteristic	CBHG + ORS (*n* = 29)	ORS (*n* = 34)	Total (*n* = 63)	*p*
**Sex, no. (%)**				0.079
Male	20 (69.0)	16 (47.1)	36 (57.1)	
Female	9 (31.0)	18 (52.9)	27 (42.9)	
**Age—years**				0.62
Average	3.1	3.2	3.15	
Median	2.0	2.0	2.0	
Standard deviation	2.8	2.3	2.5	
Interquartile interval	1.3–4.0	1.2–5.0	1.2–5.0	
Range	0.8–12.0	1.0–9.0	0.8–12.0	
**Vomiting, no. (%)**				0.739
Yes	23 (79.3)	25 (75.7)	48 (77.4)	
No	6 (20.7)	8 (24.2)	14 (22.6)	
**Fever, no. (%)**				0.121
Yes	4 (13.8)	10 (30.3)	14 (22.6)	
No	25 (86.2)	23 (69.7)	48 (77.4)	
**No. of stools**				0.112
Average	5.8	4.7	5.2	
Median	6.0	4.0	5.0	
Standard deviation	2.7	2.0	2.4	
Interquartile interval	4.0–8.0	3.0–6.0	3.0–7.0	
Range	1.0–12.0	2.0–10.0	1.0–12.0	

CBHG: colloidal bismuth hydroxide gel; ORS: oral rehydration solution.

**Table 2:** Variables observed after the treatment according to study group.

Characteristic	CBHG + ORS (*n* = 29)	ORS (*n* = 34)	Difference of Averages	*p*
			CI of 95%	
**Duration of the diarrhea (hours)**			16.0 (2.0; 34.0)	0.015
Average	25.5	41.5		
Median	22.0	24.0		
Standard deviation	28.7	40.4		
Interquartile interval	6.0–26.0	20.0–48.0		
Range	0.0–120.0	4.0–192.0		
**No. of stools**			3.4 (0.5; 7.4)	0.032
	4.8	8.2		
	3.0	4.5		
	5.7	9.3		
	1.0–6.0	2.0–9.0		
	0.0–23.0	1.0–38.0		
**Vomiting 24 h, no. (%)**			–	0.342
	7 (24.1)	5 (14.7)		
	22 (75.9)	29 (85.3)		
**Vomiting 48 h, no. (%)**			–	0.423
	3 (10.3)	2 (5.9)		
	26 (89.7)	32 (94.1)		
**Vomiting 72 h, no. (%)**			–	0.713
	1 (3.5)	1 (2.9)		
	28 (96.5)	33 (97.1)		
**Vomiting 5 days, no. (%)**			–	0.54
	0 (0.0)	1 (2.9)		
	29 (100.0)	33 (97.1)		

CBHG: colloidal bismuth hydroxide gel; ORS: oral rehydration solution; CI: confidence interval.

## Supplementary Materials

The following are available online at http://www.mdpi.com/2227-9067/3/4/45/s1, Supplementary data 1: Ethical Approval, Supplementary data 2: Informed Consent.
